# Genome-wide association analysis of chronic lymphocytic leukaemia, Hodgkin lymphoma and multiple myeloma identifies pleiotropic risk loci

**DOI:** 10.1038/srep41071

**Published:** 2017-01-23

**Authors:** Philip J. Law, Amit Sud, Jonathan S. Mitchell, Marc Henrion, Giulia Orlando, Oleg Lenive, Peter Broderick, Helen E. Speedy, David C. Johnson, Martin Kaiser, Niels Weinhold, Rosie Cooke, Nicola J. Sunter, Graham H. Jackson, Geoffrey Summerfield, Robert J. Harris, Andrew R. Pettitt, David J. Allsup, Jonathan Carmichael, James R. Bailey, Guy Pratt, Thahira Rahman, Chris Pepper, Chris Fegan, Elke Pogge von Strandmann, Andreas Engert, Asta Försti, Bowang Chen, Miguel Inacio da Silva Filho, Hauke Thomsen, Per Hoffmann, Markus M. Noethen, Lewin Eisele, Karl-Heinz Jöckel, James M. Allan, Anthony J. Swerdlow, Hartmut Goldschmidt, Daniel Catovsky, Gareth J. Morgan, Kari Hemminki, Richard S. Houlston

**Affiliations:** 1 Division of Genetics and Epidemiology, The Institute of Cancer Research, London, UK; 2 Division of Molecular Pathology, The Institute of Cancer Research, London, UK; 3 Myeloma Institute for Research and Therapy, University of Arkansas for Medical Sciences, Little Rock, USA; 4 Northern Institute for Cancer Research, Newcastle University, Newcastle upon Tyne, UK; 5 Department of Haematology, Royal Victoria Infirmary, Newcastle upon Tyne, UK; 6 Department of Haematology, Queen Elizabeth Hospital, Gateshead, Newcastle upon Tyne, UK; 7 Department of Molecular and Clinical Cancer Medicine, University of Liverpool, Liverpool, UK; 8 Queens Centre for Haematology and Oncology, Castle Hill Hospital, Hull and East Yorkshire NHS Trust, UK; 9 Department of Haematology, Birmingham Heartlands Hospital, Birmingham, UK; 10 Department of Haematology, School of Medicine, Cardiff University, Cardiff, UK; 11 Cardiff and Vale National Health Service Trust, Heath Park, Cardiff, UK; 12 Department of Internal Medicine, University Hospital of Cologne, Cologne, Germany; 13 Division of Molecular Genetic Epidemiology, German Cancer Research Centre, Heidelberg, Germany; 14 Centre for Primary Health Care Research, Lund University, Malmö, Sweden; 15 Institute of Human Genetics, University of Bonn, Germany; 16 Division of Medical Genetics, Department of Biomedicine, University of Basel, Switzerland; 17 Department of Genomics, Life & Brain Center, University of Bonn, Germany; 18 University of Duisburg–Essen, Essen, Germany; 19 Division of Breast Cancer Research, The Institute of Cancer Research, London, UK; 20 Department of Internal Medicine V, University of Heidelberg, Heidelberg, Germany; 21 National Center of Tumor Diseases, Heidelberg, Germany

## Abstract

B-cell malignancies (BCM) originate from the same cell of origin, but at different maturation stages and have distinct clinical phenotypes. Although genetic risk variants for individual BCMs have been identified, an agnostic, genome-wide search for shared genetic susceptibility has not been performed. We explored genome-wide association studies of chronic lymphocytic leukaemia (CLL, N = 1,842), Hodgkin lymphoma (HL, N = 1,465) and multiple myeloma (MM, N = 3,790). We identified a novel pleiotropic risk locus at 3q22.2 (*NCK1*, rs11715604, *P* = 1.60 × 10^−9^) with opposing effects between CLL (*P* = 1.97 × 10^−8^) and HL (*P* = 3.31 × 10^−3^). Eight established non-HLA risk loci showed pleiotropic associations. Within the HLA region, Ser37 + Phe37 in HLA-DRB1 (*P* = 1.84 × 10^−12^) was associated with increased CLL and HL risk (*P* = 4.68 × 10^−12^), and reduced MM risk (*P* = 1.12 × 10^−2^), and Gly70 in HLA-DQB1 (*P* = 3.15 × 10^−10^) showed opposing effects between CLL (*P* = 3.52 × 10^−3^) and HL (*P* = 3.41 × 10^−9^). By integrating eQTL, Hi-C and ChIP-seq data, we show that the pleiotropic risk loci are enriched for B-cell regulatory elements, as well as an over-representation of binding of key B-cell transcription factors. These data identify shared biological pathways influencing the development of CLL, HL and MM. The identification of these risk loci furthers our understanding of the aetiological basis of BCMs.

Differing in their clinical phenotype, chronic lymphocytic leukaemia (CLL), Hodgkin lymphoma (HL), and multiple myeloma (MM) are all malignancies resulting from the unrestrained clonal expansion of B-cells at different stages of maturation^1^,^2^,^3^,^4^. Evidence for inherited genetic susceptibility to CLL, HL and MM has been provided by studies of familial risk, and more recently from genome-wide association studies (GWAS) which have identified risk SNPs for each tumour type^5^,^6^,^7^,^8^,^9^,^10^,^11^,^12^,^13^,^14^,^15^,^16^,^17^,^18^,^19^,^20^. While the familial risks for CLL, HL and MM are primarily tumour-specific^21^,^22^, there is some epidemiological evidence for shared susceptibility^23^,^24^,^25^,^26^. An example of this is provided by the pattern of familial risks associated with B-cell malignancies (BCMs) in Swedish populations^25^.

Genetic variation at a number of loci, such as 5p15 and 8q24, have been shown to influence the risk of a number of BCM and non-haematological cancers^27^,^28^,^29^,^30^,^31^,^32^,^33^,^34^. Identifying risk loci that can have such pleiotropic effects is important for gaining insight into shared and divergent molecular basis of different tumour types.

While conventional meta-analysis provides a powerful tool for combining distinct GWAS, this approach is suboptimal in the presence of disease heterogeneity, such as when SNP associations are only manifest in a specific subset of the diseases, or have opposing effects for different diseases. To address such shortcomings in searching for pleiotropic risk SNPs for BCM, we adopted the previously validated association analysis based on subsets (ASSET) meta-analytic approach^35^,^36^. ASSET implements an agnostic analysis exploring all possible subsets of studies to identify the strongest association signal, while accounting for the multiple tests required by the subset search, as well as any shared controls between studies. In doing so, ASSET is able to identify variants that are positively and negatively associated with different diseases.

Applying this statistical procedure to six BCM GWAS (two each of CLL, HL and MM) we report the identification of a novel pleiotropic region influencing BCM risk, as well as eight non-HLA linked pleiotropic loci that have only previously been described in single GWAS. Within the HLA region, we report two novel coding variants in class II HLA proteins which have pleiotropic effects on BCM risk.

## Results

**Figure 1:**
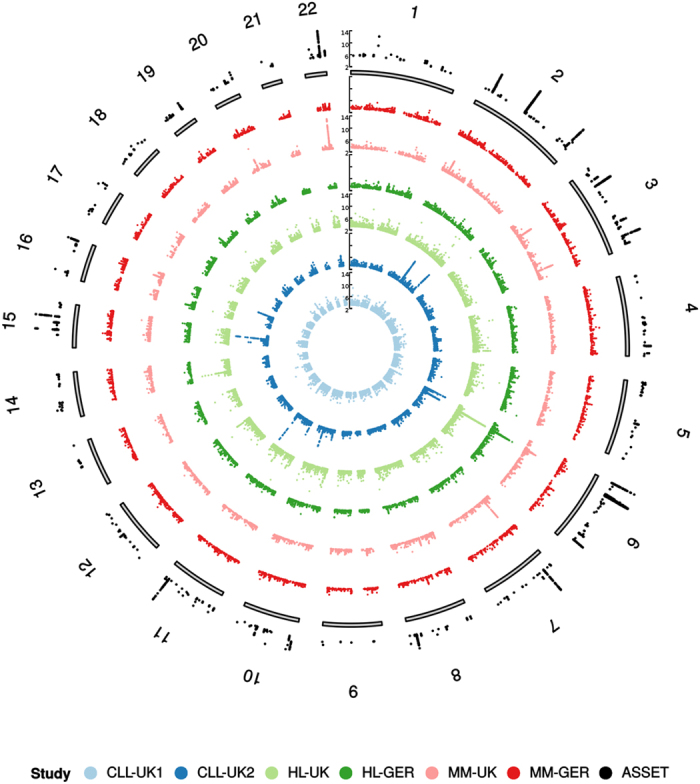
Manhattan plots (−log_10_(*P*)) by chromosome. Innermost to outermost ring – chronic lymphocytic leukaemia (CLL)-UK1, CLL-UK2, Hodgkin lymphoma (HL)-UK, HL-GER, multiple myeloma (MM)-UK, MM-GER, and ASSET association test. For clarity, only data with *P* < 1 × 10^−3^ are shown.

Characteristics of the six GWAS are summarised in Supplementary Table 1. After applying quality control filters and imputation of GWAS data (see methods) we analysed over 10 million variants for pleiotropic associations in 7,097 BCM cases and 7,324 controls of European ancestry. Figure 1 shows a Manhattan plot of the association test results for CLL, HL and MM.

To determine whether the global pleiotropic regions of association for pairs of BCMs occurred more often than expected by chance, we generated stratified quantile-quantile (Q-Q) plots to assess enrichment of associations for a given tumour type conditioned on the *P*-value for another tumour (Supplementary Fig. 1). The greater departure from the expected line associated with smaller *P*-values observed in the Q-Q plots provides evidence of pleiotropic effects between CLL, HL and MM^37^.

To identify the specific regions across the genome that demonstrate pleiotropic effects on risk of BCM we used ASSET^35^,^36^. In order for candidate SNPs to be considered, they were required to meet the following criteria: (1) variant associations at *P* ≤ 5.0 × 10^−8^ for the ASSET test; (2) at least one other variant in the same region (within *r*^2^ > 0.2) with the same pleiotropic association at *P* ≤ 1.0 × 10^−6^; (3) the individual one-sided ASSET subset tests were significant at *P* < 0.01; (4) the variant is not driven by a single study; (5) the variant cannot be both positively and negatively associated in different datasets of the same BCM; and (6) if a variant is positively and negatively associated with different BCM, the 2-sided *P*-value must be lower than both individual 1-sided *P*-values. Using these criteria, we identified nine non-HLA regions (607 variants).

### Newly identified pleiotropic risk loci

**Figure 2:**
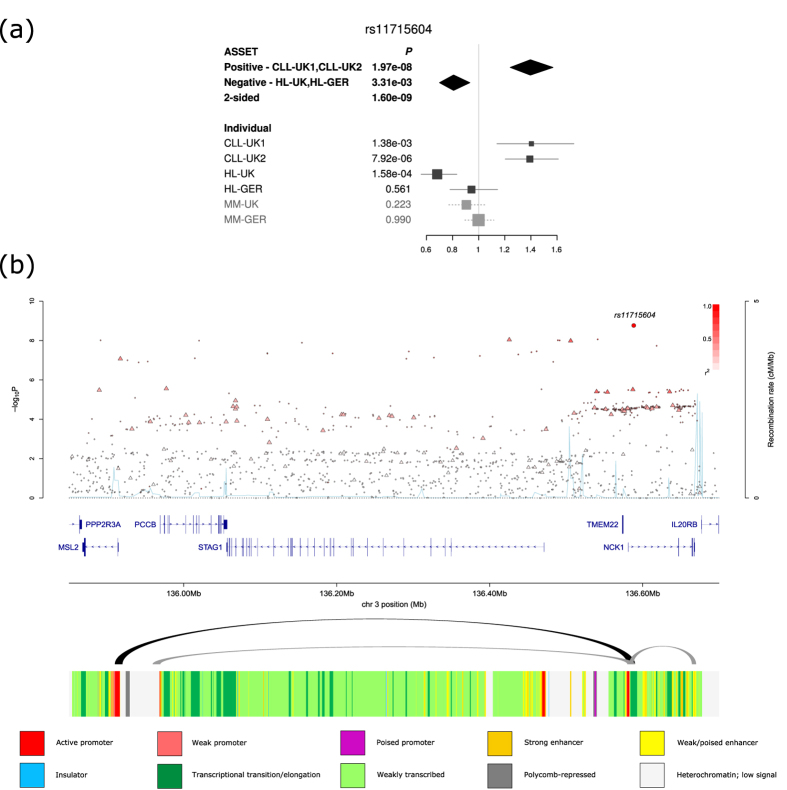
(**a**) Forest plot of the ORs for the association between rs11715604 and BCM. Studies were weighted according to the inverse of the variance of the log of the OR calculated. Horizontal lines: 95% CI. Box: OR point estimate; box area is proportional to the weight of the study. Diamond: overall summary estimate, with CI given by its width. Unbroken vertical line: null value (OR = 1.0). (**b**) Regional plot of association and recombination rates. −log_10_(*P*) (*y* axis) of the SNPs are shown according to their chromosomal positions (*x* axis). The sentinel SNP is shown as a large circle. The colour intensity of each symbol reflects the extent of LD with the sentinel SNP: white (*r*^2^ = 0) through to dark red (*r*^2^ = 1.0). Genetic recombination rates, estimated from the 1000 Genomes Project, are shown with a light blue line. Physical positions are based on NCBI build 37 of the human genome. Also shown are the relative positions of genes and transcripts mapping to the region of association. The arcs represent Hi-C promoter contacts in GM12878 cells. The colour intensity of each contact reflects the interaction score. The bottom track represents the chromatin-state segmentation track (ChromHMM) for lymphoblastoid cells using data from the HapMap ENCODE Project.

We identified a novel pleiotropic association at 3q22.2 (rs11715604, *P* = 1.60 × 10^−9^, Fig. 2) with opposing associations in CLL (*P*_*1-tailed*_ = 1.97 × 10^−8^) and HL (*P*_*1-tailed*_ = 3.31 × 10^−3^). rs11715604 maps to intron 1 of *NCK1,* which is integral to T-cell activation^38^,^39^ and regulates the PI3K/Akt pathway^40^. We also identified a number of promising associations that did not reach genome-wide significance, but exhibited moderate effects in the different BCM (Supplementary Table 2). These included associations at 22q13.33 (rs131821, *P* = 7.49 × 10^−8^) and 18p11.31 (rs634212, *P* = 5.11 × 10^−5^). rs131821 is intronic of *NCAPH2,* which is important in mitotic chromosome architecture^41^, while rs634212 is intronic of *L3MBTL4*, which has been implicated as a tumour suppressor gene for breast cancer^42^.

### Previously known risk loci with newly identified pleiotropic effects

We identified genome-wide significant pleiotropic associations (*i.e. P* ≤ 5.0 × 10^−8^) at eight non-HLA linked loci previously identified as risk factors for CLL, HL or MM (Table 1). The CLL risk loci at 6p21.32 (*BAK1*) and 6p25.3 (*IRF4*) were positively associated with HL risk^11^,^17^. In contrast the 2q13 (*BCL2L11*) and 11q24.1 (*GRAMD1B*) risk loci for CLL negatively influenced MM risk^5^,^11^. The MM risk locus at 3p22.1 (*ULK4*) positively influenced HL^7^, whereas the 2p23.3 (*DTNB*) risk locus for MM negatively associated with CLL^7^. The HL risk locus at 3p24.1 (*EOMES*) was positively associated with CLL^14^. The 3q26.2 (*TERC*) a risk factor for MM and CLL, showed a positive association with HL risk^10^,^16^. In addition to variation at these eight regions we observed promising pleiotropic associations at 2q37.1 (*SP110*, rs150468793; rs149207840)^9^, 3q27 (*LPP*, rs4459895)^6^, 5q15 (*ELL2*, rs2546191)^18^, 8q24.21 (*PVT1*, rs2720680)^12^, 15q15.1 (*BMF*, rs35603048)^5^, and 16q24.2 (*IRF8*, rs4240807)^9^ (Table 1).

**Figure 3:**
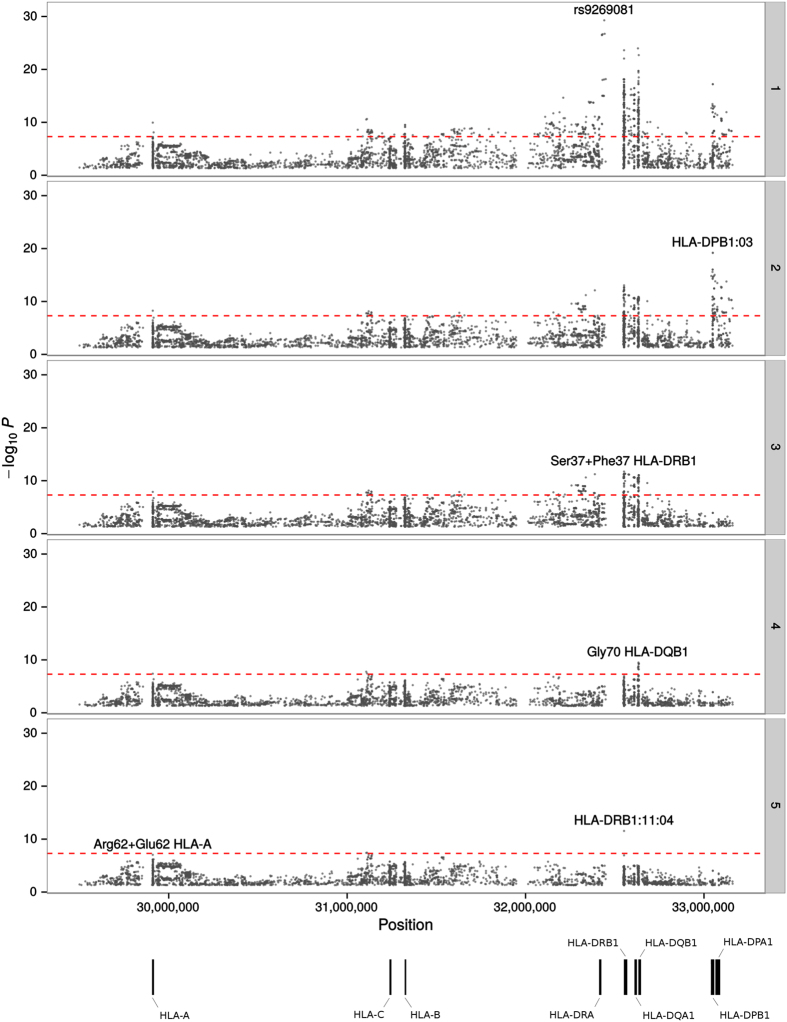
Manhattan plot representation of the step-wise conditional logistic regression of risk of BCM in the HLA region. (1) Unconditioned test of the HLA region. (2) Results of the HLA region after conditioning on rs9269081. (3) Results of the HLA region after conditioning on rs9269081 and HLA-DPB1:03. (4) Results of the HLA region after conditioning on rs9269081, HLA-DPB1:03 and Ser37 + Phe37 HLA-DRB1. (5) Results of the HLA region after conditioning on rs9269081, HLA-DPB1:03, Ser37 + Phe37 HLA-DRB1 and Gly70 HLADQB-1. The −log_10_(*P*) of the combined logistic regression test *P*-values are plotted against their physical chromosomal position. The broken red line represents the genome-wide level of significance (*P* < 5 × 10^−8^).

Association studies of CLL, HL and MM have demonstrated seemingly different associations between loci within the HLA region and risk^10^,^43^,^44^. To implement an ASSET analysis of the HLA region, we imputed classical alleles, coding variants of HLA proteins, and SNPs using the SNP2HLA software in conjunction with the Type 1 Diabetes Genetics Consortium (T1DGC) HLA reference panel^45^. Figure 3 shows the unconditioned ASSET associations across the 3.7 Mb HLA region. 768 variants demonstrated an association for CLL, HL and MM at *P* ≤ 5.0 × 10^−8^. To isolate independent pleiotropic associations we performed conditional stepwise logistic regression conditioning on the strongest associated variant from the 2-sided ASSET analysis. We identified Ser37 + Phe37 in HLA-DRB1 (*P*_*conditional*_ = 1.84 × 10^−12^), positively associated with CLL and HL (*P*_*1-tailed-conditional*_ = 4.68 × 10^−12^) and negatively associated with MM (*P*_*1-tailed-conditional*_ = 1.2 × 10^−2^). In addition, Gly70 HLA-DQB1 (*P*_*conditional*_ = 3.15 × 10^−10^) was positively associated with CLL (*P*_*1-tailed-conditional*_ = 3.52 × 10^−3^) but negatively associated with HL (*P*_*1-tailed-conditional*_ = 3.41 × 10^−9^). Additionally, a promising association for Arg62 + Glu62 in HLA-A (*P*_*conditional*_ = 9.26 × 10^−8^) was found, and was positively associated with CLL (*P*_*1-tailed-conditional*_ = 8.06 × 10^−5^) but negatively associated with HL (*P*_*1-tailed-conditional*_ = 5.68 × 10^−5^).

### Biological inference of pleiotropic risk loci

To explore whether the identified SNPs are eQTLs, we searched the Blood eQTL browser^46^, and MuTHER^47^ and Geuvadis/1000 Genomes^48^ lymphoblastoid cell line (LCL) datasets. In addition we examined expression data from MM plasma cells^49^. We found evidence for eQTLs (FDR adjusted *P* < 0.05) for nine of the pleiotropic loci in the LCL data, and four loci in the plasma cell data (Supplementary Table 3).

Since spatial proximity between specific genomic regions and chromatin looping interactions are central for regulation of gene expression^50^, we identified patterns of chromatin interactions at candidate pleiotropic SNPs by analysing Hi-C data on GM12878, as a source of B-cell information (Fig. 2 and Supplementary Fig. 2). Looping chromatin interactions were shown at 3q22 (rs11715604), 3p24 (rs9880772), 3q26 (rs12638862), 6p21 (rs210143) and 11q24 (rs4525246). The looping interactions at 3q22, implicates IL-20RB which regulates antigen-specific T-cell responses^51^. Furthermore, at 3p24, we observed looping interactions with *AZI2*, which contributes to the activation of NF-κB^52^.

Across the BCM pleiotropic risk loci, we confirmed enrichment of regulatory elements in primary haematopoietic stem cells (*P* = 2.1 × 10^−3^) and GM12878 cells (*P* = 7.4 × 10^−3^, Supplementary Table 4)^53^. Analysis of ChIP-seq data on 82 transcription factors (TFs) showed an enrichment of binding of key B-cell transcription factors, including CEBPB, RXRA, and POLR3G (*P* < 0.05/82 = 6.10 × 10^−4^) (Supplementary Fig. 3). CEBPB is a TF that is involved in immune and inflammatory responses^54^, and can induce reprogramming of B-cells into pluripotent stem cells^55^. RXRA can induce B-cell differentiation^56^, and POLR3G is a DNA-dependent RNA polymerase III^57^.

### Pathway analysis and construction of a susceptibility network

We performed a gene-set enrichment analysis to gain insight into the biological pathways perturbed by genetic variation common to CLL, HL and MM, and found eight pathways related to the inflammatory response and antigen processing that showed enrichment (*i.e.* FDR adjusted *P*-value < 0.05; Supplementary Table 5). Following on from this analysis, we investigated the inter-connectivity of the associated genes^58^. By constructing a network of published and predicted protein-protein interactions, protein co-localisations and protein domain similarity, we delineated two broad clusters – one related to BCL2, and the other related to HLA (Supplementary Fig. 4).

## Discussion

Motivated by the stratified Q-Q plots, which suggested the existence of pleiotropy, we utilised genotype data from six datasets in British and German populations, to conduct an agnostic cross-cancer genome-wide analysis to identify specific pleiotropic associations for CLL, HL and MM for both HLA and non-HLA regions. We identified a number of promising associations that have strong biological plausibility, including *NCK1, NCAPH2* and *L3MBTL4.* A contemporaneous analysis also used ASSET across a number of different non-BCM cancers, and discovered a novel risk locus at 1q22 involving breast and lung cancer^59^.

Our analysis also provides evidence for common and opposing effects being responsible for BCM pathogenesis, but is not the first to identify opposing risk associations in different cancers^60^,^61^. Given that many of the identified risk loci harbour genes integral for immune function, it is entirely conceivable that balancing selection may act to ensure immune diversity and thus a selective advantage against temporal environmental risk factors such as infection^62^.

As with standard GWAS analyses ASSET may not identify the causative genetic variant at a locus. Accepting this caveat, many of the identified regions map to eQTL and regulatory elements in B-cells. Moreover, they feature an over-representation of key B-cell TF binding.

The HLA class II region has previously been implicated in multiple BCM including follicular lymphoma^33^,^63^, HL^12^ and CLL^17^,^44^. Here, we additionally show the involvement of this region in the development of MM. By performing a more refined imputation analysis on the HLA region, we found a variant that alters amino acid 37 of HLA-DRB1. This change affects the electrostatic properties of the P9 binding pocket^64^, altering T-cell receptor recognition^65^. The second pleiotropic association at HLA region at amino acid 70 of HLA-DQB1 is located in the P4 binding pocket, which is also a critical residue influencing antigen T-cell receptor binding^66^. A previous study of a number of different B-cell lymphomas using over 7,000 cases also found an association in the HLA region^67^, further highlighting the importance of this region to the development of BCM. In addition to the HLA association, we identified other associations that were independently ascertained in the BCM specific GWAS, including 3p24.1 (*EOMES*) for HL^14^ and CLL^6^, and 3q26.2 (*TERC*) for MM^10^ and CLL^16^, thus adding confidence that ASSET method is able to identify common genetic components.

Although predicated on protein-protein interactions, our pathway analysis provides two core cellular functions influencing BCM susceptibility. Firstly, antigen presentation/T-cell regulation, centred around HLA, and secondly cellular growth and apoptosis, centred around BCL2, which are interconnected through the key B-cell regulators, MYB and GATA3^68^,^69^. *BCL2* is commonly overexpressed in BCM and is relevant to tumour escape apoptosis^70^,^71^,^72^. It is noteworthy that Venetoclax, a BCL2 inhibitor used in treatment of CLL^73^, may also be efficacious in treating other forms of BCM^74^. This exemplifies that targeting pathways identified through GWAS may inform drug discovery initiatives^75^.

In conclusion, using data from six GWAS we have identified associations with multiple BCM. There are likely additional loci that have an effect, but their detection will require additional efforts with larger datasets. Such future analyses should also address the disparity in sample sizes of each of the BCM series that characterises our study.

## Methods

### Subjects and GWAS datasets

We used data generated from GWAS of CLL, HL, and MM performed in European populations which have been the subject of previous publications^10^,^11^,^12^,^14^,^16^. Briefly, the MM-UK GWAS comprised 2,282 cases (1,060 male; mean age at diagnosis: 64 years) recruited through the UK Medical Research Council (MRC) Myeloma-IX and Myeloma-XI trials. The MM-GER GWAS comprised 1,508 cases (867 male; mean age at diagnosis: 59 years) recruited by the German Multiple Myeloma Study Group (GMMMG) coordinated by the University Clinic, Heidelberg. The HL-UK GWAS comprised 622 cases ascertained through: (i) the Royal Marsden Hospital National Health Service Trust Family History study during 2004–2008 (*n* = 104, 63 male; mean age at diagnosis: 38 years); and (ii) an ongoing national study of HL in females (*n* = 518, mean age at diagnosis: 23 years) conducted by the Institute of Cancer Research (ICR). The HL-GER GWAS comprised 1,001 HL cases (597 male; mean age at diagnosis: 35 years) ascertained by the German Hodgkin Study Group during 1998–2007. The CLL-UK1 GWAS comprised 517 cases: (i) 155 cases (95 male; mean age at diagnosis: 59 years) from ICLLLC; and (ii) 362 cases (269 male; mean age at diagnosis: 63 years) from the Leukaemia Research CLL-4 trial. CLL-UK2 comprised 1,403 cases collected from two ongoing initiatives: (i) 1,111 cases collected through a UK national study of CLL genetics coordinated by the ICR (712 male; mean age at diagnosis: 63 years); and (ii) 292 cases collected through the Newcastle CLL Consortium (181 male; mean age at diagnosis: 66 years) from patients attending six haematology units in the UK.

Collection of blood samples and clinical information from subjects was undertaken with informed written consent and relevant ethical review board approval at respective institutions, in accordance with the tenets of the Declaration of Helsinki. Specifically, approval for the CLL data was approved by the UK Multi-Research Ethics Committee (MREC 99/1/082). For the MM data, the Myeloma-IX trial was approved by the Medical Research Council Leukaemia Data Monitoring and Ethics committee (MREC 02/8/95, ISRCTN68454111), the Myeloma-XI trial by the Oxfordshire Research Ethics Committee (MREC 17/09/09, ISRCTN49407852), and the GMMMG study was approved by the University of Heidelberg Ethical Commission (229/2003, S-337/2009, AFmu-119/2010). For the HL data, approval was obtained from the Multi-Research Ethics Committee (MREC 03/1/096) for the UK data, and the Ethics Committee of the University of Cologne for the German data. All methods and experimental protocols were performed in accordance with relevant guidelines and regulations.

Genotyping of cases was performed using Illumina arrays: CLL-UK1 on 317 K array, HL-UK on 660w-Quad BeadChip, and CLL-UK2, HL-GER and all MM samples using Omni-express arrays (Illumina, San Diego, CA, US). For the UK controls, we used publicly accessible data generated by the Wellcome Trust Case Control Consortium (WTCCC), the 1958 Birth Cohort (also known as the National Child Development Study) and UK Blood Service^76^. Genotyping of both sets of controls was conducted using Illumina Human 1.2M-Duo Custom_v1 Array BeadChips. For the German studies we utilised controls from the Heinz Nixdorf Recall study genotyped using Illumina OmniExpress array^77^.

Full details of the genotyping of cases and quality control can be found in previously published work^10^,^14^,^16^. Briefly, general genotyping quality control assessment was as previously described^78^ and all SNPs presented in this study passed the required thresholds. Duplicate samples were used to check genotyping quality. SNPs and samples with <95% SNPs genotyped were eliminated from the analyses. Genotype frequencies at each SNP were tested for deviation from the Hardy–Weinberg equilibrium and rejected at *P* < 10^−5^. The number of samples and variants that passed quality control is provided in Supplementary Table 1.

We have previously confirmed an absence of systematic genetic differences between cases and controls^10^,^14^,^16^. Prediction of the untyped SNPs was carried out using IMPUTEv2 based on a merged reference panel from UK10K (April 2014 release) and from the 1000 Genomes Project (phase 1 v3)^79^,^80^. Association meta-analyses only included markers with info scores > 0.4, imputed call rates/SNP > 0.9 and MAFs > 0.005.

### Statistical analysis

The association between variants with cancer risk in each of the six GWAS was evaluated by logistic regression under a log additive model using SNPTEST v2^79^. In the MM-GER study, genomic inflation due to population stratification was detected (λ > 1.1), so the per-allele odds ratios (ORs) were adjusted using principal components obtained from smartPCA^81^.

To investigate pleiotropy globally, we generated stratified Q-Q plots of association signals in one cancer stratified by the *P*-values in a second cancer^37^, for every combination of BCM. Leftward inflation in the null line is indicative of a higher degree of pleiotropy between the two tumours than expected by chance.

Subset meta-analysis was conducted using the R statistical package ASSET (association analysis based on subsets) which explores all possible subsets of “non-null” studies to identify the strongest association signal and then evaluates the significance of the signal while accounting for multiple tests required by the subset search^35^,^36^. One-tailed tests are subsequently combined to produce a 2-sided test statistic. Although ASSET has the advantage of accounting for subsets of studies with no effects and/or effects in opposing directions, where a large majority of effects are in one direction it will have lower power compared to the conventional fixed-effect analysis. The number of overlapping subjects in the GWAS (*i.e.* controls from WTCCC^76^ and Heinz-Nixdorf^77^ controls) were used as a covariate when estimating standard errors^35^. Imputed SNPs that showed significant associations were genotyped using standardised Sanger sequencing methods to confirm the imputation fidelity.

### HLA imputation and analysis

To determine whether specific coding variants within HLA genes contributed to the diverse association signals, we imputed the classical HLA alleles (A, B, C, DQA1, DQB1, DRB1) and coding variants across the HLA region using SNP2HLA^45^. The imputation was based on a reference panel from the Type 1 Diabetes Genetics Consortium (T1DGC) consisting of genotype data from 5,225 individuals of European descent with genotyping data of 8,961 common SNPs and indel polymorphisms across the HLA region, and four digit genotyping data of the HLA class I and II molecules. This reference panel has been used previously and showed high imputation quality for the HLA region in other studies^45^,^82^,^83^.

To identify independent effects, dependency analyses by step-wise logistic regression were carried out by conditioning on the strongest association signal in the specific BCM. The index SNP at each region was included as a covariate, and the association statistics were recalculated for the remaining test SNPs. This process was repeated until no SNPs reached the minimum level of significance. The criteria for declaring an independent effect were defined as *P* < 5 × 10^−8^.

### Functional prediction

LD between SNPs were calculated with VCFtools^84^ using data from the UK10K (April 2014 release) and the 1000 Genomes Project (phase 1 v3)^79^,^80^. These data were plotted using visPIG^85^.

To explore the epigenetic profile of genomic location associated with BCM, we used ENCODE histone modification data and HaploReg and RegulomeDB^86^,^87^ to examine whether any of the SNPs or their proxies (*i.e. r*^2^ > 0.8 in the 1000 Genomes EUR reference panel) annotate transcription factor binding or enhancer elements.

To examine enrichment in specific TF binding across risk loci we adapted the variant set enrichment method of Cowper-Sal lari *et al*.^88^ Briefly, for each risk locus, a region of strong LD (defined as r^2^ > 0.8 and D’ > 0.8) was determined, and these SNP were termed the associated variant set (AVS). TF ChIP-seq uniform peak data was obtained from ENCODE for the GM12878 cell line, and included data for 82 TF. For each of these marks the overlap of the SNP in the AVS and the binding sites was determined to produce a mapping tally. SNPs with the same LD structure as the risk associated SNP were randomly selected to calculate a null mapping tally. A null distribution was produced by repeating this process 10,000 times, and approximate *P*-values were calculated as the proportion of permutations where the null mapping tally was greater or equal to the AVS mapping tally. An enrichment score was calculated by normalising the tallies to the median of the null distribution. Thus the enrichment score is the number of standard deviations of the AVS mapping tally from the mean of the null distribution tallies.

### eQTL analysis

The presence of potential eQTL was investigated through the use of several public data sets, namely the Blood eQTL browser^46^ in whole blood, and MuTHER^47^ and Geuvadis/1000 Genomes^48^ in lymphoblastoid cell lines (LCL).

For myeloma plasma cell eQTL analysis, we included a German (n = 658) and a UK (n = 183) case series which had been the subject of a previous eQTL analysis^49^ and 608 cases of a recently published US GWAS 13. Gene expression profiling of CD138-purified plasma cells using Affymetrix U133 2.0 plus arrays was performed as described^89^,^90^,^91^. Pre-processing of expression data was done as previously published^49^. Briefly, we used the Affymetrix U133 2.0 plus array custom (CDF) (v17) mapping to Entrez genes^92^ as chip definition file and excluded microarray probes binding to polymorphic sites. Expression data were normalized using GC-RMA. We only included genes with log_2_ expression > 3.5 in at least 95% of samples of each set. After quality control and excluding autosomal genes, expression data for 8,505 genes was available. The filtered set was analysed using probabilistic estimation of expression residuals (PEER)^93^ to infer known and hidden intervening variables, such as cytogenetic subgroups.

For the Geuvadis and MM plasma cell data, the relationship between SNPs and expression of genes located within 1 Mb was analysed using the Matrix eQTL^94^ package under a linear model. In all the datasets, SNPs in LD (*r*^2^ > 0.8) with the potential pleiotropic associations were explored, and were included where FDR adjusted *P* < 0.05.

### Network analysis

Pathway enrichment analysis was performed using the Improved Gene Set Enrichment Analysis for Genome-wide Association Study (i-GSEA4GWAS v2)^95^. This tool also performed a functional annotation analysis on these pathways by identifying the top SNPs that map to the pathway genes, and determining if any of these SNPs fall within ENCODE peak data, namely DNase-seq peaks of open chromatin, FAIRE peaks of open chromatin, TFBS SPP-based peaks, TFBS PeakSeq-based peaks and Histone peaks. In addition, eQTLs were determined using several eQTL databases, namely eQTL Browser, GTEx and seeQTL. Common networks were identified using GeneMANIA^58^. This database collated data on protein and genetic interactions from a number of sources, including BioGRID, InterPro, Reactome, and Ensembl.

### Promoter capture Hi-C data

To map risk SNP to interaction involving promoter contacts and identify genes involved in HL susceptibility, we analysed previously published promoter capture Hi-C data on the GM12878 cell line as a model B-cell^96^. The promoter capture Hi-C interactions were used to functionally annotate GWAS SNPs to seek for evidences of looping between the SNPs and the promoters of nearby genes. Reads from technical replicates were combined before processing and valid pairs were identified using HICUP^97^. Two biological replicates were analysed to assure reproducibility and significant interactions were determined using CHiCAGO^98^.

## Additional Information

**How to cite this article**: Law, P. J. *et al*. Genome-wide association analysis of chronic lymphocytic leukaemia, Hodgkin lymphoma and multiple myeloma identifies pleiotropic risk loci. *Sci. Rep.* **7**, 41071; doi: 10.1038/srep41071 (2017).

**Publisher's note:** Springer Nature remains neutral with regard to jurisdictional claims in published maps and institutional affiliations.

## Supplementary Material

Supplementary Data

Supplementary Table 4

## Figures and Tables

**Table 1:** Novel pleiotropic associations in genomic regions already identified through single disease genome-wide association studies.

Locus	SNP ID	Position (bp)	Allele 1	Allele 2	ASSET 2-sided *P*-value	Disease Group 1			Disease Group 2		
						BCM	Odds Ratio (CI)	*P*-value	BCM	Odds Ratio (CI)	*P*-value
2p23.3	rs6546149	25629438	C	G	6.27 × 10^−10^	CLL	1.09 (1.01–1.17)	2.14 × 10^−2^	**MM**	0.83 (0.78–0.88)	1.15 × 10^−9^
2q13	rs12711846	111856293	A	G	3.48 × 10^−14^	**CLL**	1.44 (1.31–1.58)	6.37 × 10^−14^	MM	0.92 (0.86–0.98)	1.53 × 10^−2^
3p24.1	rs9880772	27777779	G	A	7.42 × 10^−9^	CLL, **HL**	1.18 (1.11–1.24)	7.42 × 10^−9^			
3p22.1	rs6763508	41750989	T	C	7.56 × 10^−12^	**MM**, HL	1.22 (1.16–1.30)	7.56 × 10^−12^			
3q26.2	rs12638862	169477506	G	A	1.88 × 10^−11^	**CLL**, **MM**, HL	1.15 (1.09–1.19)	1.88 × 10^−11^			
6p25.3	rs9392017	442357	A	G	6.03 × 10^−9^	**CLL**, HL	1.22 (1.15–1.30)	6.03 × 10^−9^			
6p21.32	rs210143	33546837	T	C	6.81 × 10^−12^	**CLL**, HL	1.24 (1.17–1.32)	6.81 × 10^−12^			
11q24.1	rs4525246	123395246	G	C	3.37 × 10^−14^	**CLL**	1.40 (1.28–1.53)	6.33 × 10^−14^	MM	0.93 (0.87–0.99)	1.50 × 10^−2^
2q37.1	rs150468793; rs149207840	231144578	T	TCCTCCTG	9.63 × 10^−8^	**CLL**, MM	1.16 (1.10–1.22)	9.63 × 10^−8^			
3q27.3	rs4459895	187954414	A	C	1.70 × 10^−7^	**CLL**	1.12 (1.02–1.23)	1.35 × 10^−2^	HL	0.76 (0.68–0.85)	6.43 × 10^−7^
5q15	rs2546191	95232541	G	A	4.15 × 10^−7^	HL	1.18 (1.07–1.29)	5.38 × 10^−4^	**MM**	0.88 (0.82–0.93)	4.14 × 10^−5^
8q24.21	rs2720680	129115217	A	G	6.78 × 10^−8^	**HL**	1.27 (1.16–1.39)	1.13 × 10^−7^	CLL	0.92 (0.86–0.99)	2.9 × 10^−2^
15q15.1	rs35603048	40391965	C	T	3.64 × 10^−7^	HL	1.17 (1.06–1.28)	1.23 × 10^−3^	**CLL**	0.81 (0.74–0.89)	1.58 × 10^−5^
16q24.2	rs4240807	85985361	A	C	6.62 × 10^−7^	**CLL**	1.21 (1.11–1.32)	1.35 × 10^−5^	HL	0.86 (0.79–0.95)	2.70 × 10^−3^

Genome-wide significant associations are shown above, and promising associations are below. B-cell malignancies (BCM) in bold indicate the disease the SNP was previously shown to be associated. Odds ratio calculated from allele 2.
