# Supplementary material for: Genome-wide association analysis of chronic lymphocytic leukaemia, Hodgkin lymphoma and multiple myeloma identifies pleiotropic risk loci
Source: Sci Rep. 2017 Jan 23;7:41071. doi: 10.1038/srep41071 (PMC5253627; doi:10.1038/srep41071)

# Genome-wide association analysis of chronic lymphocytic leukaemia, Hodgkin lymphoma and multiple myeloma identifies pleiotropic risk loci

## Supplementary Data

Philip J Law, Amit Sud, Jonathan S Mitchell, Marc Henrion, Giulia Orlando, Oleg Lenive, Peter Broderick, Helen E Speedy, David C Johnson, Martin Kaiser, Niels Weinhold, Rosie Cooke, Nicola J Sunter, Graham H Jackson, Geoffrey Summerfield, Robert J Harris, Andrew R Pettitt, David J Allsup, Jonathan Carmichael, James R Bailey, Guy Pratt, Thahira Rahman, Chris Pepper, Chris Fegan, Elke Pogge von Strandmann, Andreas Engert, Asta Försti, Bowang Chen, Miguel Inacio da Silva Filho, Hauke Thomsen, Per Hoffmann, Markus M Noethen, Lewin Eisele, Karl-Heinz Jöckel, James M Allan, Anthony J Swerdlow, Hartmut Goldschmidt, Daniel Catovsky, Gareth J Morgan, Kari Hemminki, Richard S Houlston

**Supplementary Table 1:** Summary of the sample sets used in the study. The numbers shown are after QC measures.

| Cancer | Cases | Genotyping platform | Controls | Genotyping platform | Imputation filter | Number of variants |
|---|---|---|---|---|---|---|
| **Chronic lymphocytic leukaemia** | | | | | | |
| CLL-UK1 | 503 | Illumina Human 317K array | 2,700* | Illumina Human 1.2M-Duo Custom_v1 Array BeadChip | 0.4 | 10,472,381 |
| CLL-UK2 | 1,339 | Illumina OmniExpress BeadChip | 2,500* | Illumina Human 1.2M-Duo Custom_v1 Array BeadChip | 0.4 | 10,524,731 |
| **Multiple myeloma** | | | | | | |
| MM-UK | 2,282 | Illumina OmniExpress BeadChip | 5,197* | Illumina Human 1.2M-Duo Custom_v1 Array BeadChip | 0.4 | 10,504,468 |
| MM-GER | 1,508 | Illumina OmniExpress BeadChip | 2,107† | Illumina Human Omni1-Quad BeadChips or Illumina OmniExpress BeadChip | 0.4 | 10,539,844 |
| **Hodgkin lymphoma** | | | | | | |
| HL-UK | 589 | Illumina 660w-Quad BeadChip | 5,199* | Illumina Human 1.2M-Duo Custom_v1 Array BeadChip | 0.4 | 10,507,971 |
| HL-GER | 876 | Illumina OmniExpress BeadChip | 1,218† | Illumina OmniExpress BeadChip | 0.4 | 10,553,927 |
| **Total** | **7,097** | | **7,324‡** | | | **10,806,625±** |

* Controls comprise of the WTCCC 1958 Birth Cohort (1958BC) and National Blood Service (NBS) which are split between UK-CLL1 and UK-CLL2 respectively.
† Controls comprise of the Heinz-Nixdorf Recall study (HNR)
‡ The number of unique individuals after accounting for overlapping controls
± Number of unique variants present in at least two diseases

**Supplementary Table 2:** Table of ASSET results which showed moderate effects. BCM: B-cell malignancy. Odds-ratios (OR) were calculated based on allele 2.

| | | | | | | Disease Group 1 | | | Disease Group 2 | | |
|---|---|---|---|---|---|---|---|---|---|---|---|
| **Locus** | **SNP** | **Position (bp)** | **Allele 1** | **Allele 2** | **ASSET 2-sided *P*-value** | **BCM** | **OR** | ***P*-value** | **BCM** | **OR** | ***P*-value** |
| 1p35.2 | rs148297606 | 31862772 | G | C | $4.02x10^{-6}$ | CLL,HL | 2.09 (1.53-2.86) | $4.02x10^{-6}$ | - | - | - |
| 11q22.1 | rs4278486 | 98801158 | T | C | $1.05x10^{-5}$ | CLL | 1.16 (1.07-1.25) | $1.93x10^{-4}$ | HL | 0.88 (0.81-0.96) | $3.58x10^{-3}$ |
| 12q12 | rs10748274 | 38233264 | C | T | $9.55x10^{-6}$ | CLL,HL | 1.14 (1.08-1.21) | $9.55x10^{-6}$ | - | - | - |
| 12q21.2 | rs181181503 | 74669967 | T | C | $3.41x10^{-7}$ | CLL,HL | 2.56 (1.79-3.68) | $3.41x10^{-7}$ | - | - | - |
| 13q21.31 | rs1576377 | 61260524 | T | C | $3.58x10^{-6}$ | HL | 1.20 (1.09-1.32) | $3.37x10^{-4}$ | CLL | 0.86 (0.79-0.94) | $6.50x10^{-4}$ |
| 18p11.31 | rs634212 | 6033023 | G | T | $5.11x10^{-5}$ | CLL,HL | 1.37 (1.14-1.63) | $6.48x10^{-4}$ | MM | 0.80 (0.68-0.94) | $5.85x10^{-3}$ |
| 19p13.11 | rs73005220 | 16272689 | A | G | $1.30x10^{-6}$ | - | - | - | CLL,MM | 0.75 (0.67-0.85) | $1.30x10^{-6}$ |
| 22q12.3 | rs9306298 | 35677701 | T | C | $5.43x10^{-5}$ | CLL,HL | 1.12 (1.06-1.19) | $8.38x10^{-5}$ | MM | 0.95 (0.9-1.00) | 0.05 |
| 22q13.33 | rs131821 | 50950076 | A | AT | $7.49x10^{-8}$ | CLL,MM | 1.14 (1.09-1.19) | $7.49x10^{-8}$ | - | - | - |

**Supplementary Table 3:** Results of the eQTL analysis. All SNPs in LD with the SNP identified in the ASSET analysis were analysed for potential eQTL. The MuTHER, Blood eQTL browser and Geuvardis databases were used to investigate lymphoblastoid cells, in addition to data from myeloma plasma cells. Only FDR adjusted *P*<0.05 are shown.

| Region | ASSET SNP | Top eQTL SNP | $R^2$ | D' | eQTL Gene | MuTHER | | Blood eQTL browser | | Geuvardis | | Myeloma | |
|---|---|---|---|---|---|---|---|---|---|---|---|---|---|
| | | | | | | Probe | *FDR adjusted P* | Probe | *FDR adjusted P* | Probe | *FDR adjusted P* | Probe | *FDR adjusted P* |
| 2q13 | rs12711846 | rs2018707 | 0.80 | 0.99 | *BCL2L11* | ILMN_1774997 | $2.40x10^{-3}$ | | | | | | |
| 2q37.1 | rs150468793; rs149207840 | rs150468793; rs149207840 | 1.00 | 1.00 | *SP140* | | | | | | | 93349_at | $7.26x10^{-5}$ |
| 2p23.3 | rs6546149 | rs6546148 | 0.85 | 0.98 | *ADCY3* | ILMN_1676893 | 0.02 | | | | | | |
| 3p22.1 | rs6763508 | rs1016669 | 0.93 | 0.97 | *ULK4* | | | | | ENST00000301831 | $4.29x10^{-50}$ | | |
| 3p24.1 | rs9880772 | rs11129295 | 0.72 | 1.00 | *EOMES* | | | 7320372 | $4.54x10^{-5}$ | | | | |
| 3q22.2 | rs11715604 | rs71630059 | 0.71 | 1.00 | *NCK1-AS1* | | | | | ENST00000474250 | $1.17x10^{-8}$ | | |
| 3q26.2 | rs12638862 | rs12696304 | 0.94 | 1.00 | *LRRC31* | ILMN_1803528 | $1.81x10^{-7}$ | | | | | | |
| 5q15 | rs2546191 | rs9314162 | 0.97 | 1.00 | *ELL2* | | | | | | | 22936_at | $1.82x10^{-28}$ |
| 6p21.32 | rs210143 | rs210134 | 0.90 | 0.98 | *CUTA* | | | 1030427 | $2.20x10^{-9}$ | | | | |
| 11q24.1 | rs4525246 | rs4525246 | 1.00 | 1.00 | *VWA5A* | | | | | | | 4013_at | $2.76x10^{-3}$ |
| 15q15.1 | rs35603048 | rs35603048 | 1.00 | 1.00 | *EIF2AK4* | | | | | ENST00000263791 | 0.04 | | |
| 16q24.2 | rs4240807 | rs10863202 | 0.81 | 0.99 | *IRF8* | ILMN_1666594 | 0.02 | | | | | | |
| 22q13.33 | rs131821 | rs131821 | 1.00 | 1.00 | *TYMP* | | | | | | | 1890_at | $1.22x10^{-4}$ |

**Supplementary Table 4:** HaploReg results showing enrichment of regulatory elements in primary haematopoietic stem cells and GM12878 cells

(see separate file)

**Supplementary Table 5:** Results of gene set enrichment analysis in BCM risk using i-GSEA4GWAS v2. 8 pathways with FDR<0.05 were identified.

| | | | # Significantly enriched peaks (*P*-value<0.05) | | | | |
|---|---|---|---|---|---|---|---|
| **Pathway** | **FDR** | **eQTL *P*-value** | **DNase-seq** | **FAIRE** | **TFBS-PeakSeq** | **TFBS-SPP** | **Histone** |
| KEGG: antigen processing and presentation | 0.001 | $2.34 \times 10^{-57}$ | 70 | 7 | 87 | 57 | 128 |
| KEGG: intestinal immune network for IGA production | 0.001 | $6.40 \times 10^{-25}$ | 0 | 3 | 20 | 7 | 5 |
| BioCarta: inflam pathway | 0.001 | $1.84 \times 10^{-18}$ | 1 | 3 | 12 | 0 | 14 |
| KEGG: cell adhesion molecules cams | 0.001 | $9.04 \times 10^{-58}$ | 0 | 0 | 11 | 7 | 2 |
| BioCarta: cytokine pathwaY | 0.01 | $1.80 \times 10^{-8}$ | 0 | 3 | 9 | 0 | 11 |
| BioCarta: DC pathway | 0.01 | $6.91 \times 10^{-14}$ | 0 | 0 | 0 | 1 | 3 |
| GO: G protein signaling coupled to cAMP nucleotide second messenger | 0.01 | $2.70 \times 10^{-85}$ | 0 | 0 | 0 | 0 | 0 |
| GO: cAMP mediated signaling | 0.04 | $3.23 \times 10^{-85}$ | 0 | 0 | 0 | 0 | 0 |

**Supplementary Figure 1:** Conditional Q-Q plots of pleiotropic association in chronic lymphocytic leukaemia (CLL), Hodgkin lymphoma (HL) and multiple myeloma (MM) in the UK studies. The upward deflection of the observed data associated with smaller expected *P*-values seen in the Q-Q plots provides evidence of pleiotropic effects in CLL, HL and MM. CLL conditioned on HL and MM (upper panels). HL conditioned on CLL and MM (middle panels). MM conditioned on CLL and HL (lower panels).

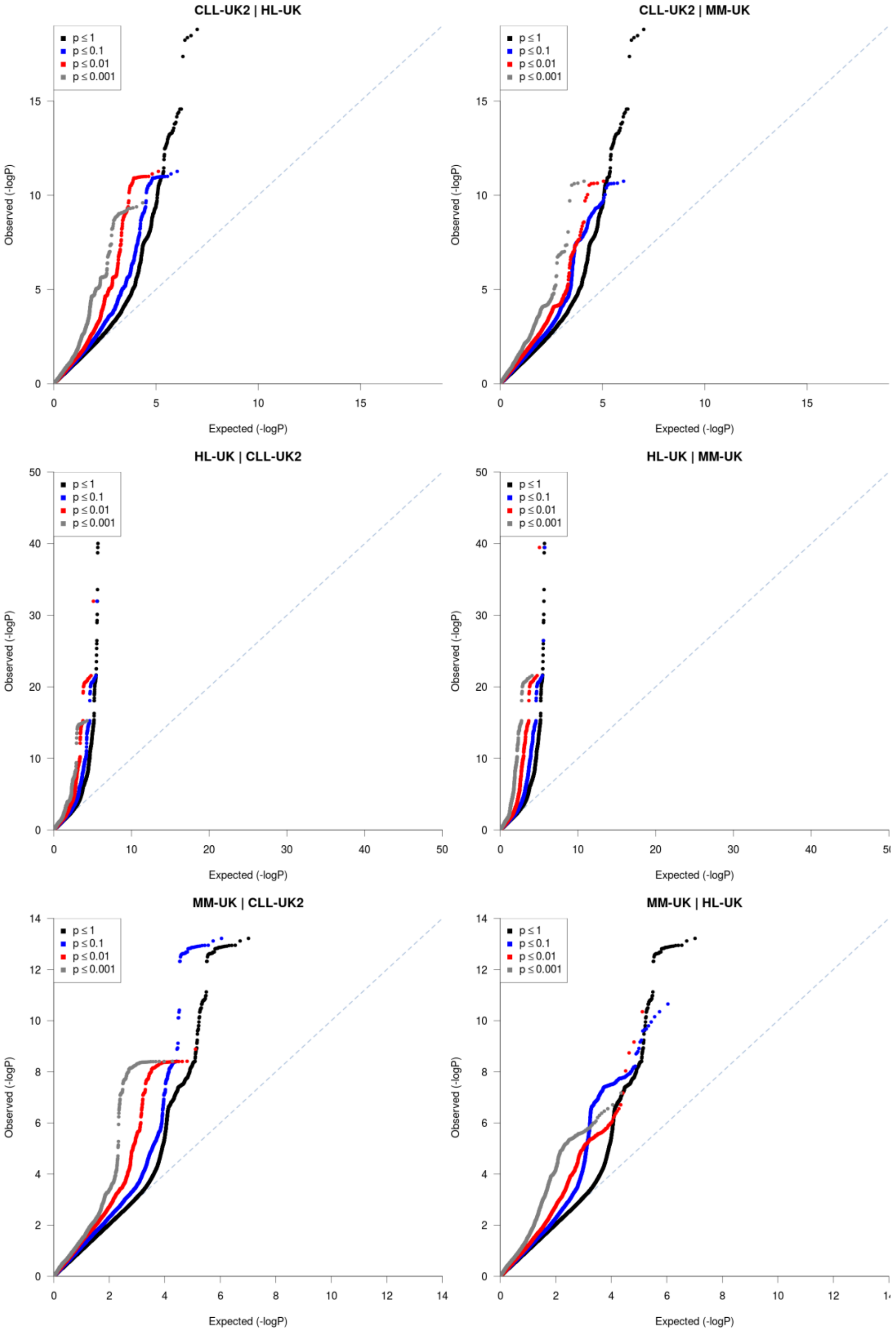

**Supplementary Figure 2**: Association plots show association results of SNPs and recombination rates. -log10(P) (y axes) of the SNPs are shown according to their chromosomal positions (x axes). The sentinel SNP is shown as a large circle. The color intensity of each symbol reflects the extent of LD with the sentinel SNP: white ($r2 = 0$) through to dark red ($r2 = 1.0$). Genetic recombination rates, estimated from the 1000 Genomes Project, are shown with a light blue line. Physical positions are based on NCBI build 37 of the human genome. Also shown are the relative positions of genes and transcripts mapping to the region of association. The middle track represents the chromatin-state segmentation track (ChromHMM) for lymphoblastoid cells using data from the HapMap ENCODE Project. The top track represents Hi-C promoter contacts in GM12878 cells. The colour intensity of each contact reflects the interaction score.

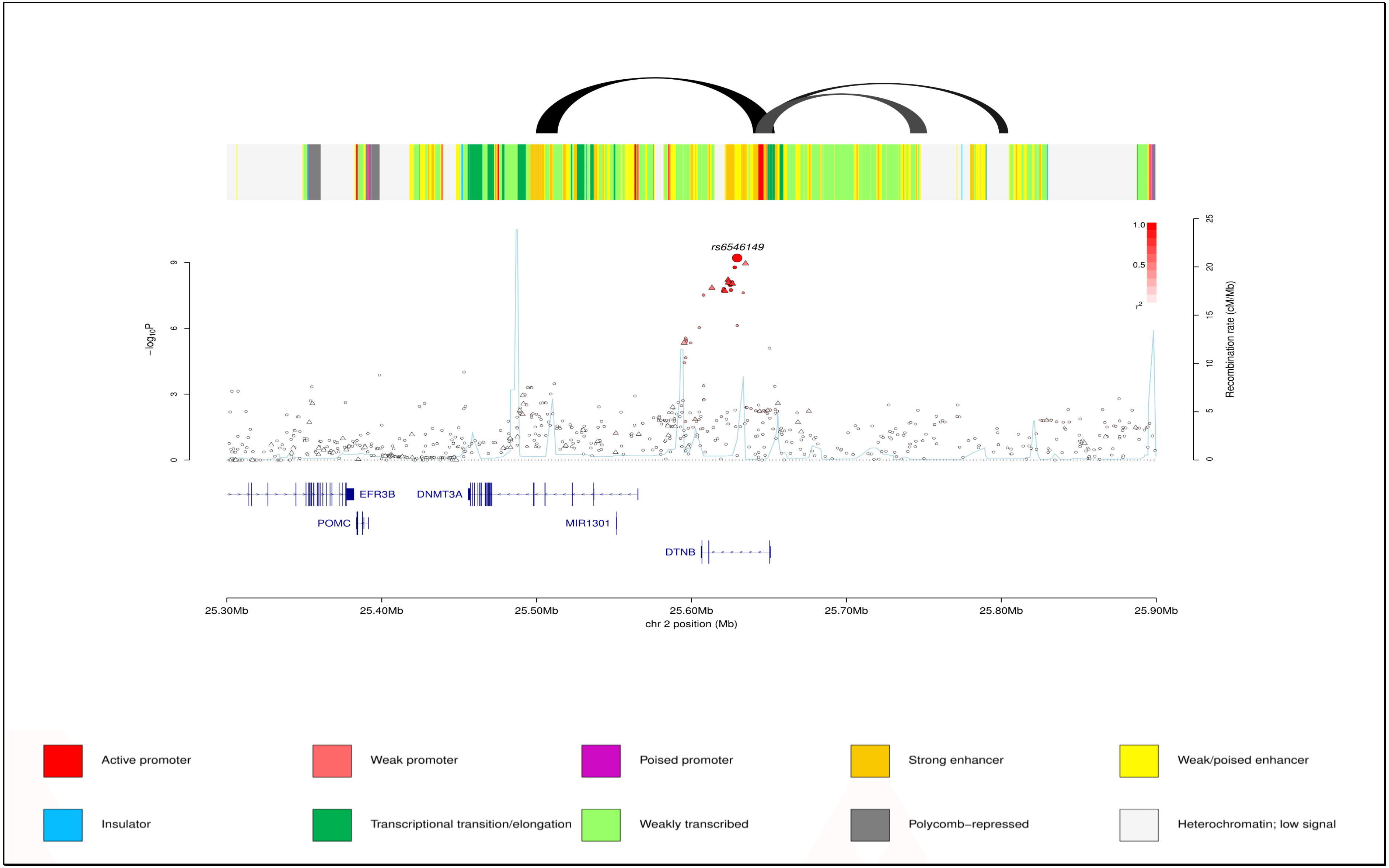
rs6546149
1.0
0.5
r²
−log10P
Recombination rate (cM/Mb)
EFR3B
DNMT3A
POMC
MIR1301
DTNB
25.30Mb
25.40Mb
25.50Mb
25.60Mb
25.70Mb
25.80Mb
25.90Mb
chr 2 position (Mb)
Active promoter
Weak promoter
Poised promoter
Strong enhancer
Weak/poised enhancer
Insulator
Transcriptional transition/elongation
Weakly transcribed
Polycomb−repressed
Heterochromatin; low signal

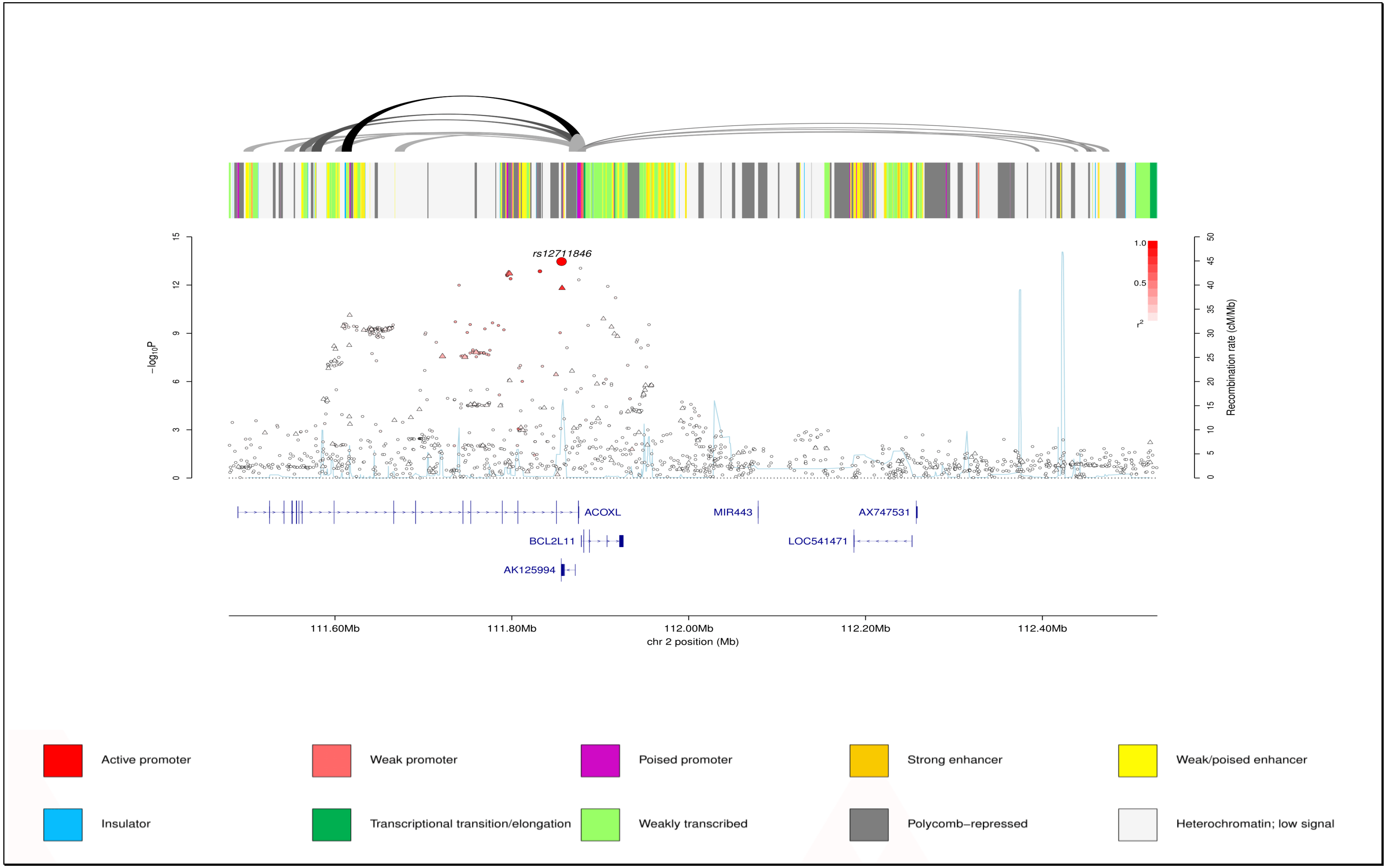

rs12711846
−log10P
Recombination rate (cM/Mb)
r2
1.0
0.5
ACOXL
MIR443
AX747531
BCL2L11
LOC541471
AK125994
111.60Mb
111.80Mb
112.00Mb
112.20Mb
112.40Mb
chr 2 position (Mb)
Active promoter
Weak promoter
Poised promoter
Strong enhancer
Weak/poised enhancer
Insulator
Transcriptional transition/elongation
Weakly transcribed
Polycomb−repressed
Heterochromatin; low signal

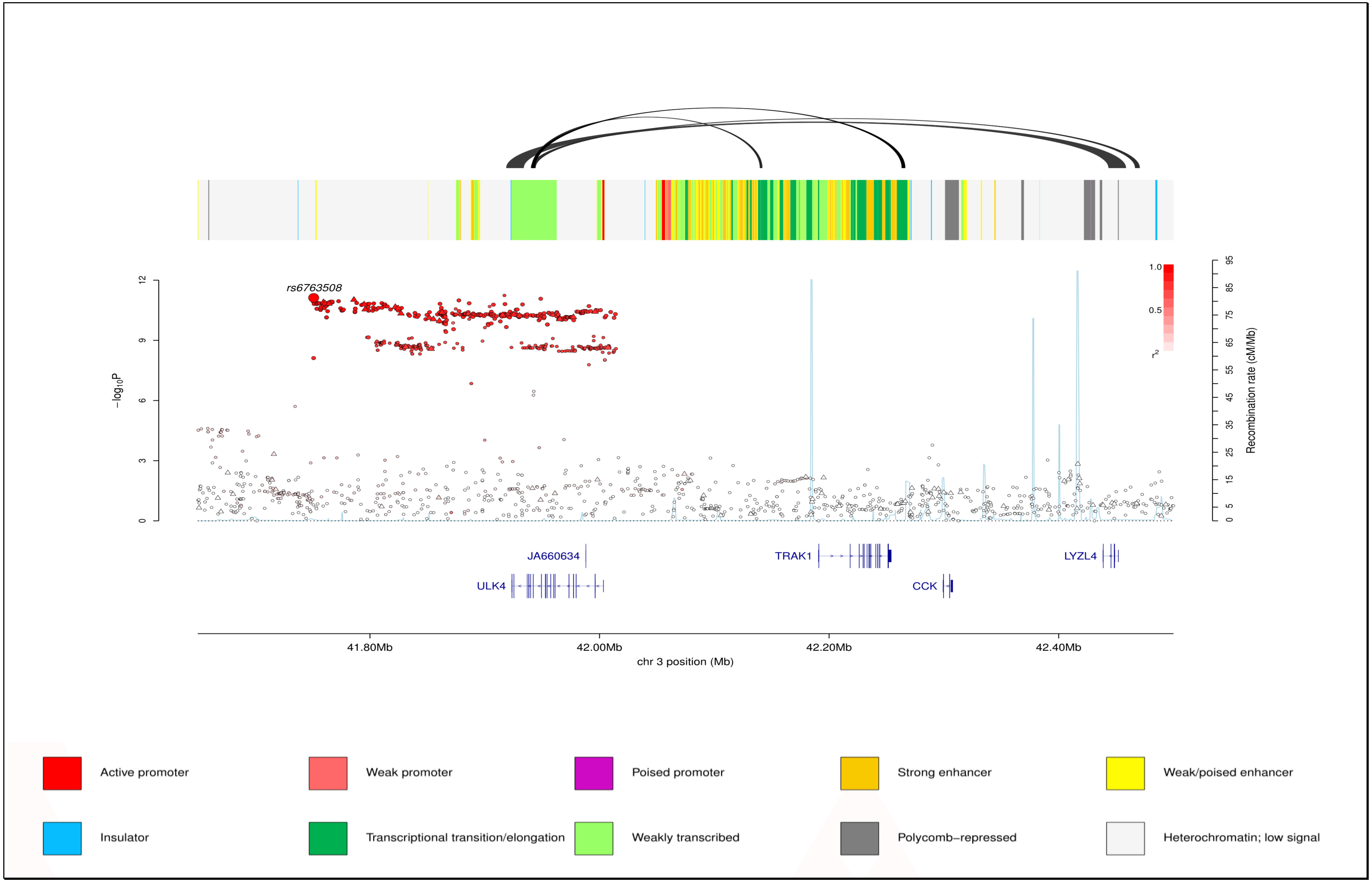

rs6763508
−log10P
Recombination rate (cM/Mb)
r2
JA660634
ULK4
TRAK1
CCK
LYZL4
41.80Mb
42.00Mb
42.20Mb
42.40Mb
chr 3 position (Mb)
Active promoter
Weak promoter
Poised promoter
Strong enhancer
Weak/poised enhancer
Insulator
Transcriptional transition/elongation
Weakly transcribed
Polycomb−repressed
Heterochromatin; low signal

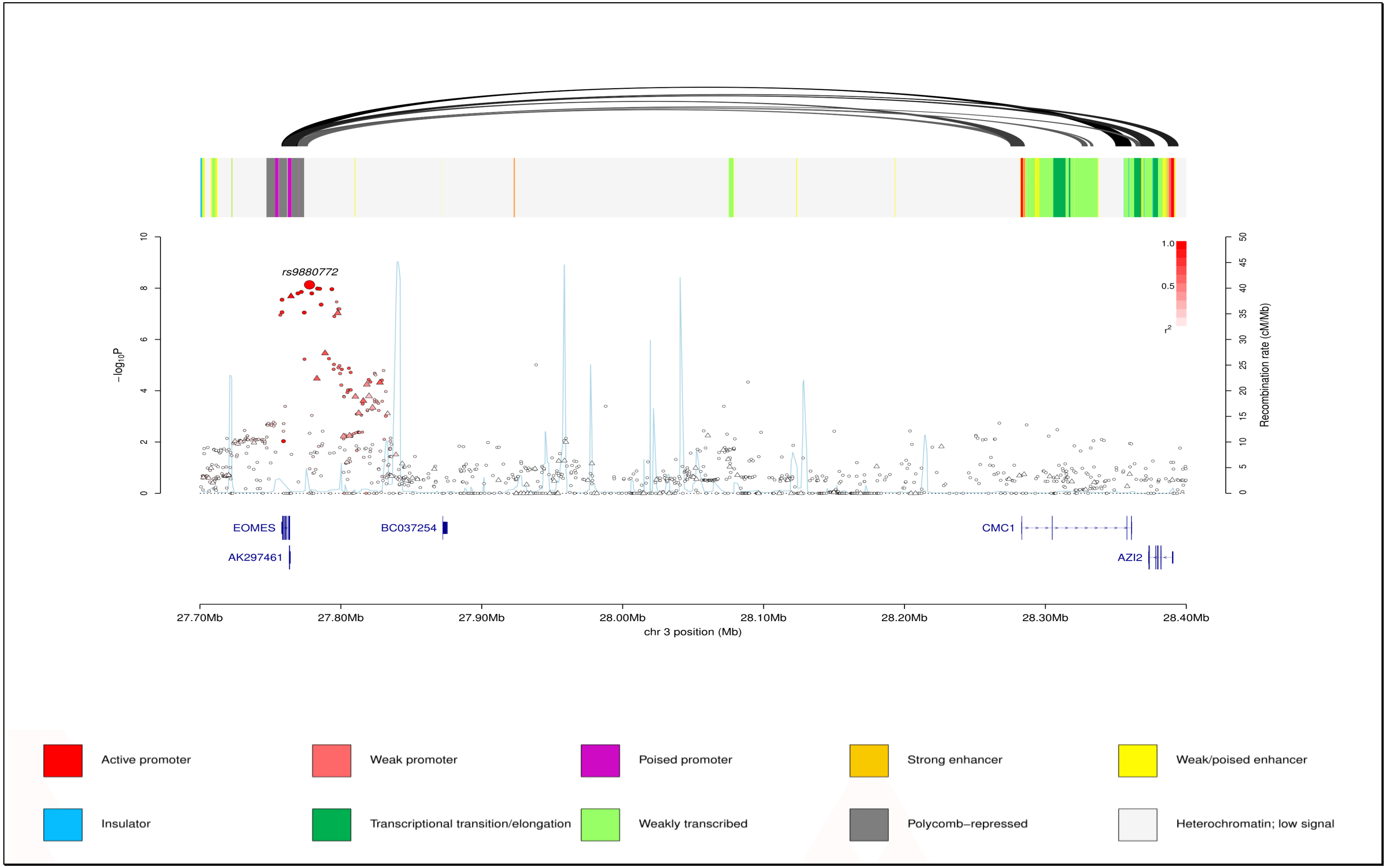
rs9880772
−log10P
Recombination rate (cM/Mb)
r2
EOMES
AK297461
BC037254
CMC1
AZI2
27.70Mb
27.80Mb
27.90Mb
28.00Mb
28.10Mb
28.20Mb
28.30Mb
28.40Mb
chr 3 position (Mb)
Active promoter
Weak promoter
Poised promoter
Strong enhancer
Weak/poised enhancer
Insulator
Transcriptional transition/elongation
Weakly transcribed
Polycomb−repressed
Heterochromatin; low signal

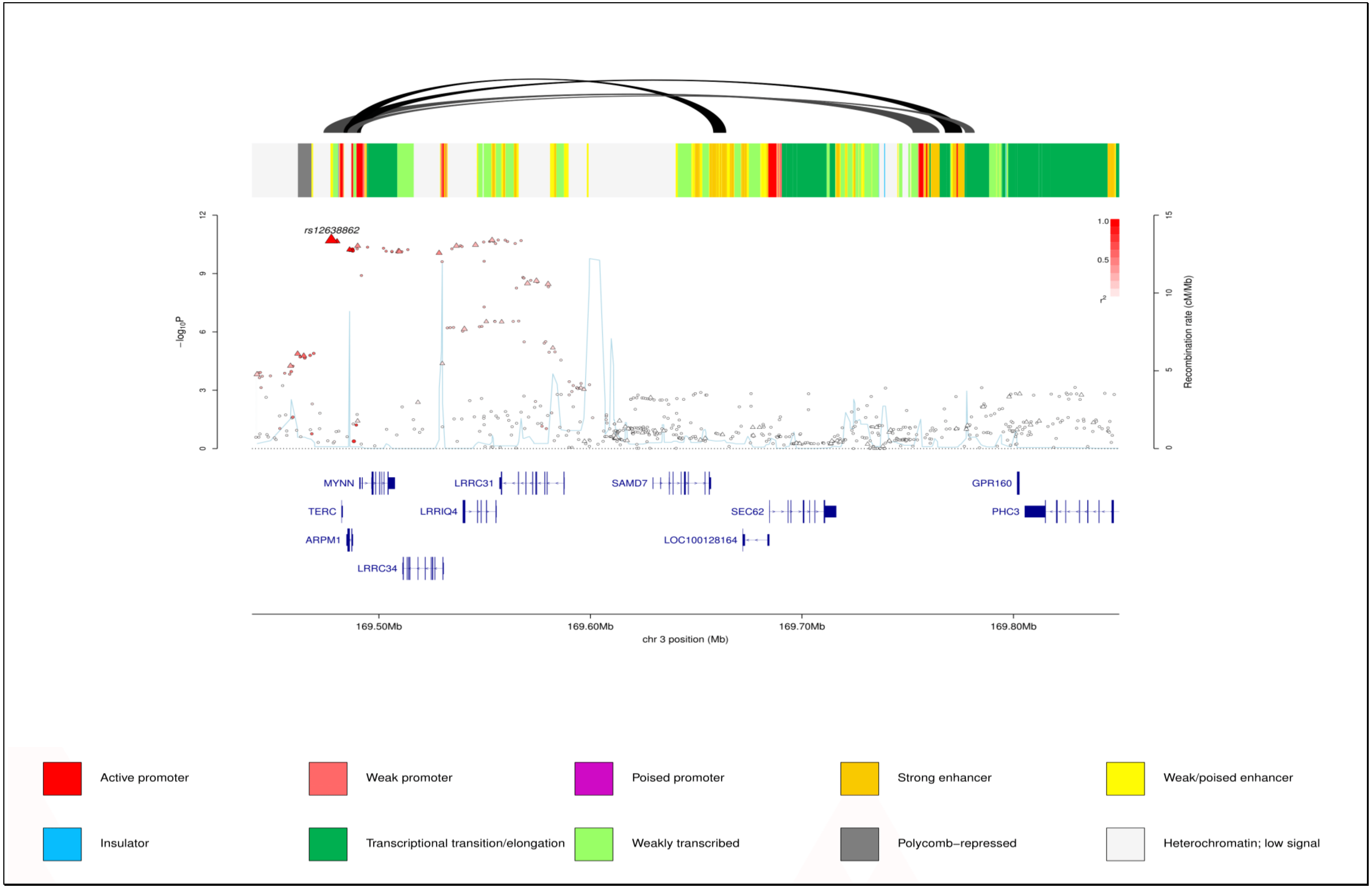
rs12638862
$-\log_{10}P$
Recombination rate (cM/Mb)
$r^2$
MYNN
TERC
ARPM1
LRRC34
LRRC31
LRRIQ4
SAMD7
SEC62
LOC100128164
GPR160
PHC3
169.50Mb
169.60Mb
169.70Mb
169.80Mb
chr 3 position (Mb)
Active promoter
Weak promoter
Poised promoter
Strong enhancer
Weak/poised enhancer
Insulator
Transcriptional transition/elongation
Weakly transcribed
Polycomb–repressed
Heterochromatin; low signal

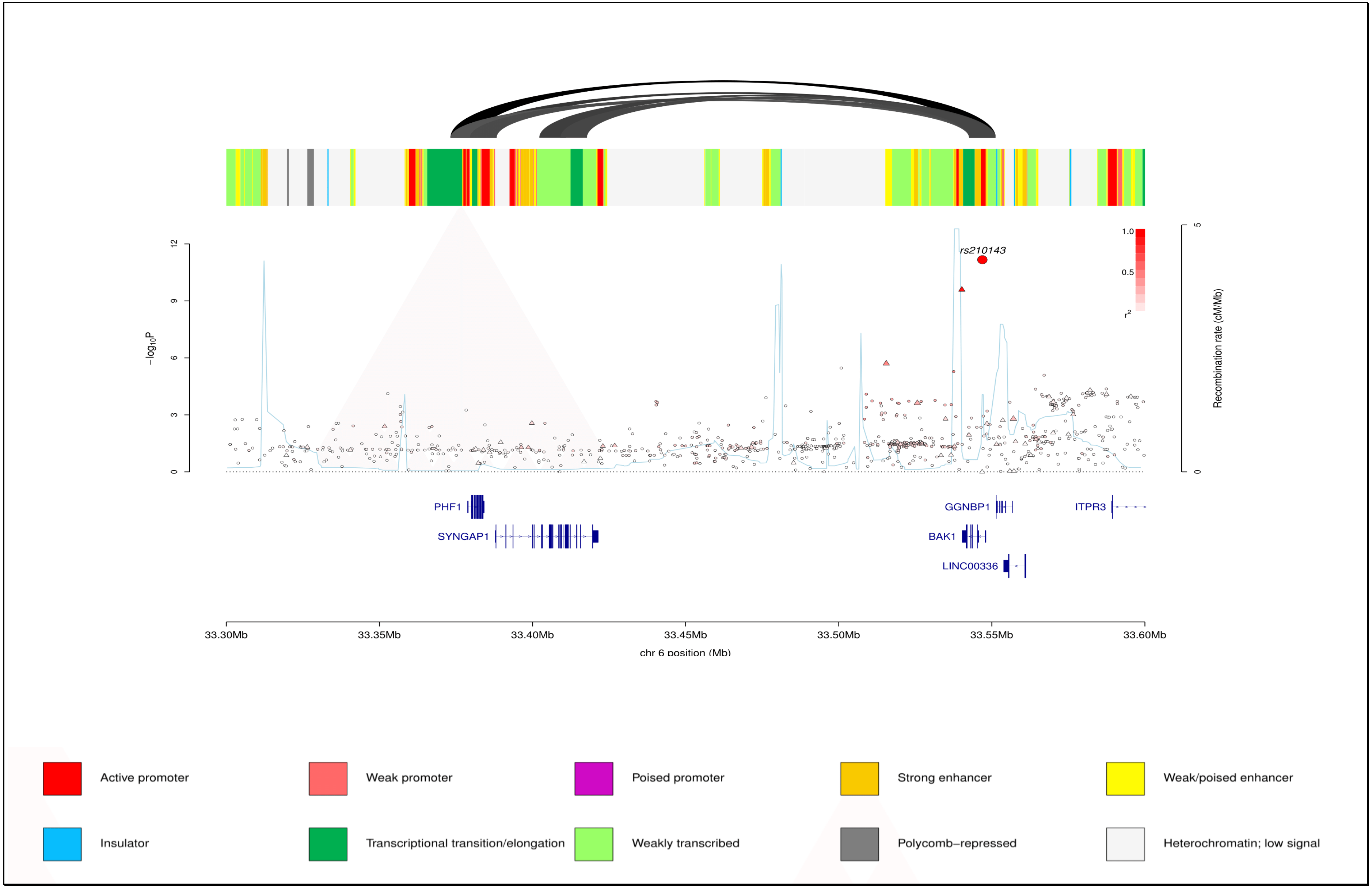

rs210143
−log10P
Recombination rate (cM/Mb)
r2
1.0
0.5
PHF1
SYNGAP1
GGNBP1
BAK1
LINC00336
ITPR3
33.30Mb
33.35Mb
33.40Mb
33.45Mb
33.50Mb
33.55Mb
33.60Mb
chr 6 position (Mb)
Active promoter
Weak promoter
Poised promoter
Strong enhancer
Weak/poised enhancer
Insulator
Transcriptional transition/elongation
Weakly transcribed
Polycomb−repressed
Heterochromatin; low signal

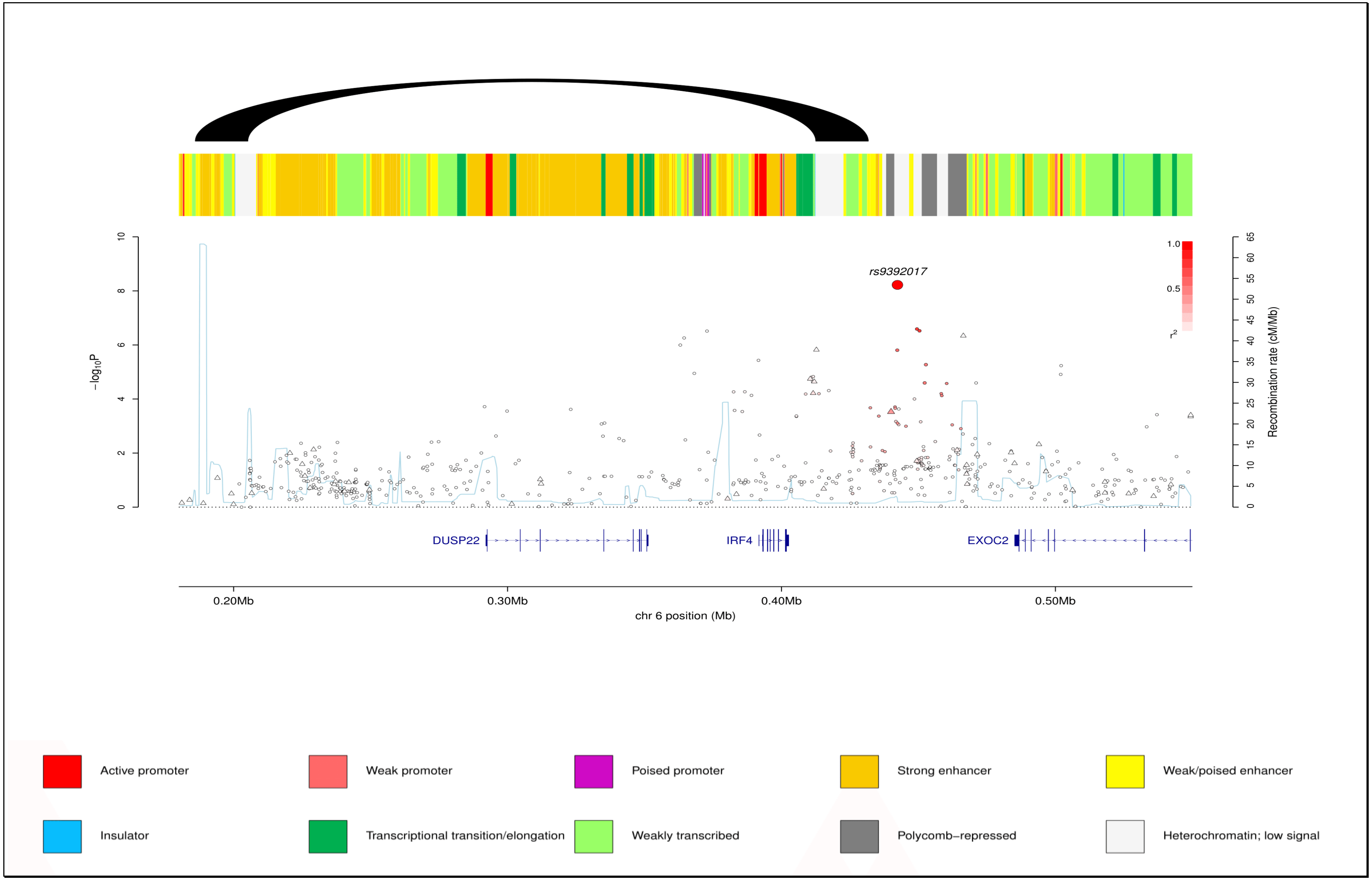

rs9392017
1.0
0.5
$r^2$
$-\log_{10}P$
Recombination rate (cM/Mb)
DUSP22
IRF4
EXOC2
0.20Mb
0.30Mb
0.40Mb
0.50Mb
chr 6 position (Mb)
Active promoter
Weak promoter
Poised promoter
Strong enhancer
Weak/poised enhancer
Insulator
Transcriptional transition/elongation
Weakly transcribed
Polycomb−repressed
Heterochromatin; low signal

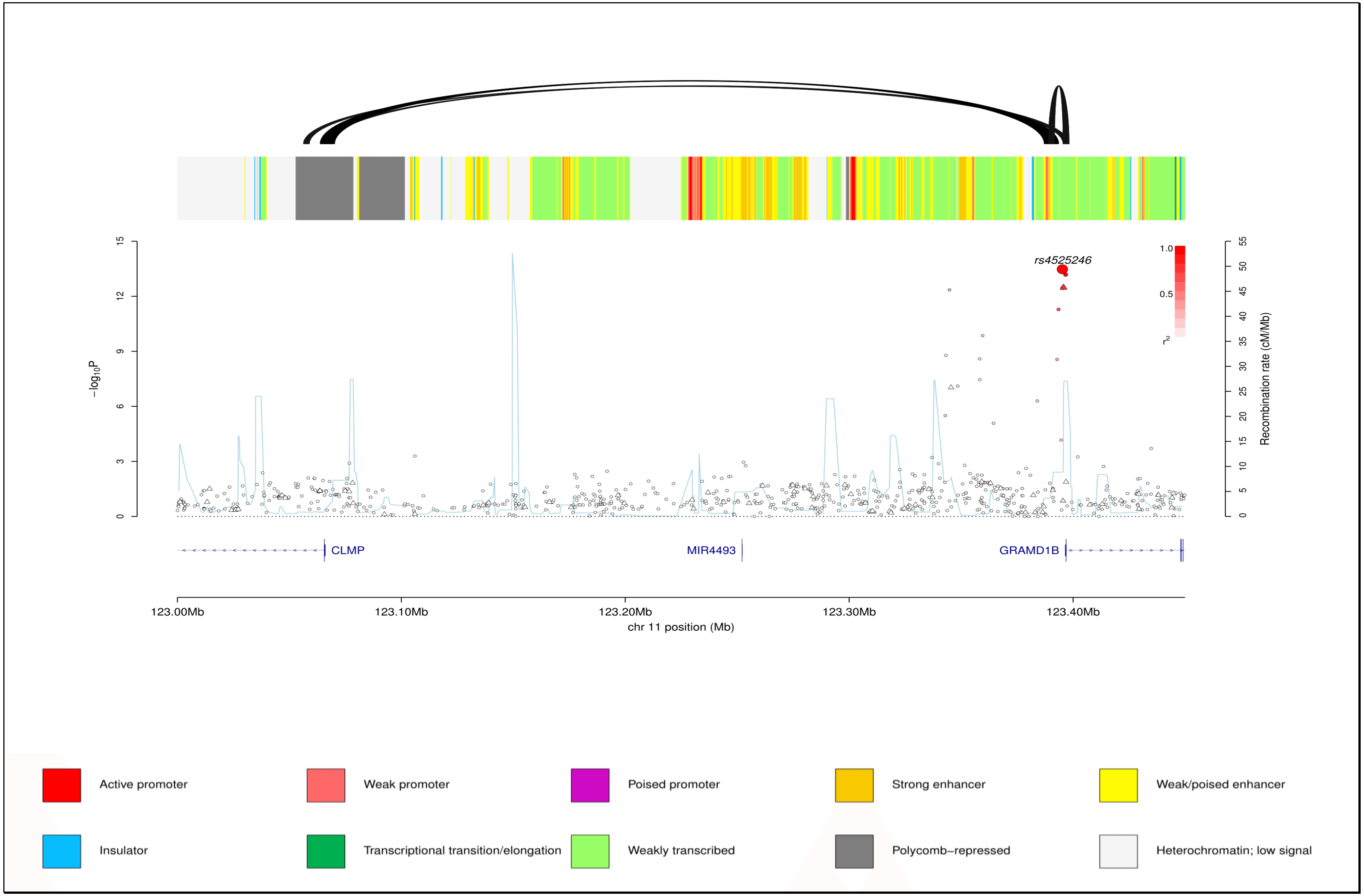

rs4525246
1.0
0.5
r²
15
12
9
6
3
0
−log10P
55
50
45
40
35
30
25
20
15
10
5
0
Recombination rate (cM/Mb)
CLMP
MIR4493
GRAMD1B
123.00Mb
123.10Mb
123.20Mb
123.30Mb
123.40Mb
chr 11 position (Mb)
Active promoter
Weak promoter
Poised promoter
Strong enhancer
Weak/poised enhancer
Insulator
Transcriptional transition/elongation
Weakly transcribed
Polycomb−repressed
Heterochromatin; low signal

**Supplementary Figure 3**: Enrichment of transcription factors and histone marks using the variants that exhibited evidence of pleiotropy at a genome-wide significant level. The red line represents the Bonferroni corrected *P*-value threshold.

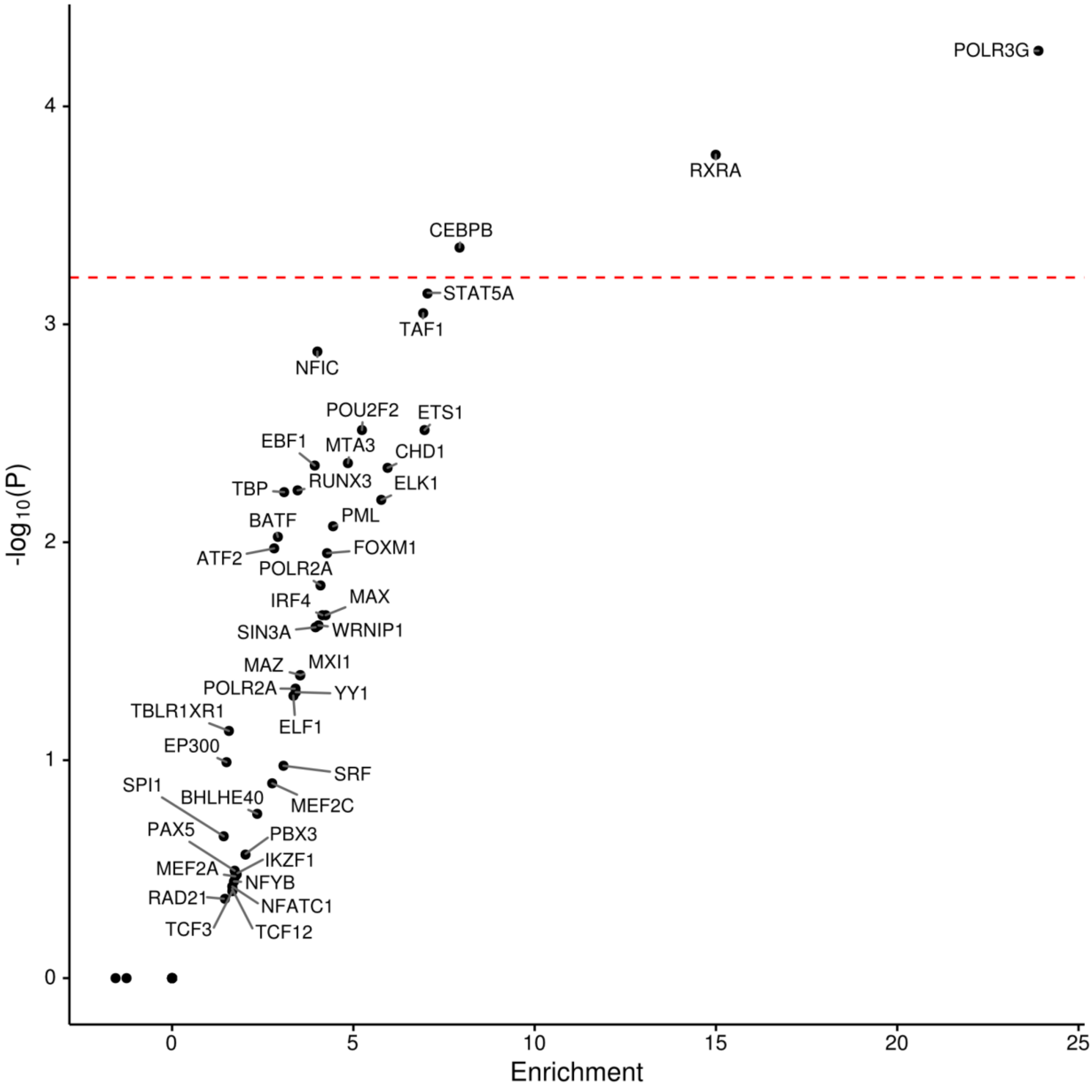

**Supplementary Figure 4:** Network analysis demonstrating the interactions between the protein product of genes at loci where variants are associated with chronic lymphocytic leukaemia (CLL), Hodgkin lymphoma (HL) and multiple myeloma (MM). Proteins are represented by coloured boxes: red, novel pleiotropic loci; gold, CLL; green, HL; purple, MM. Interaction types are represented by different line colours: orange, colocalisation; green, pathway; black, physical interaction; grey, predicted; blue, shared protein domains. Edge thickness is weighted by the confidence of the prediction. Networks were identified using GeneMANIA, and the final graph was edited using Cytoscape.

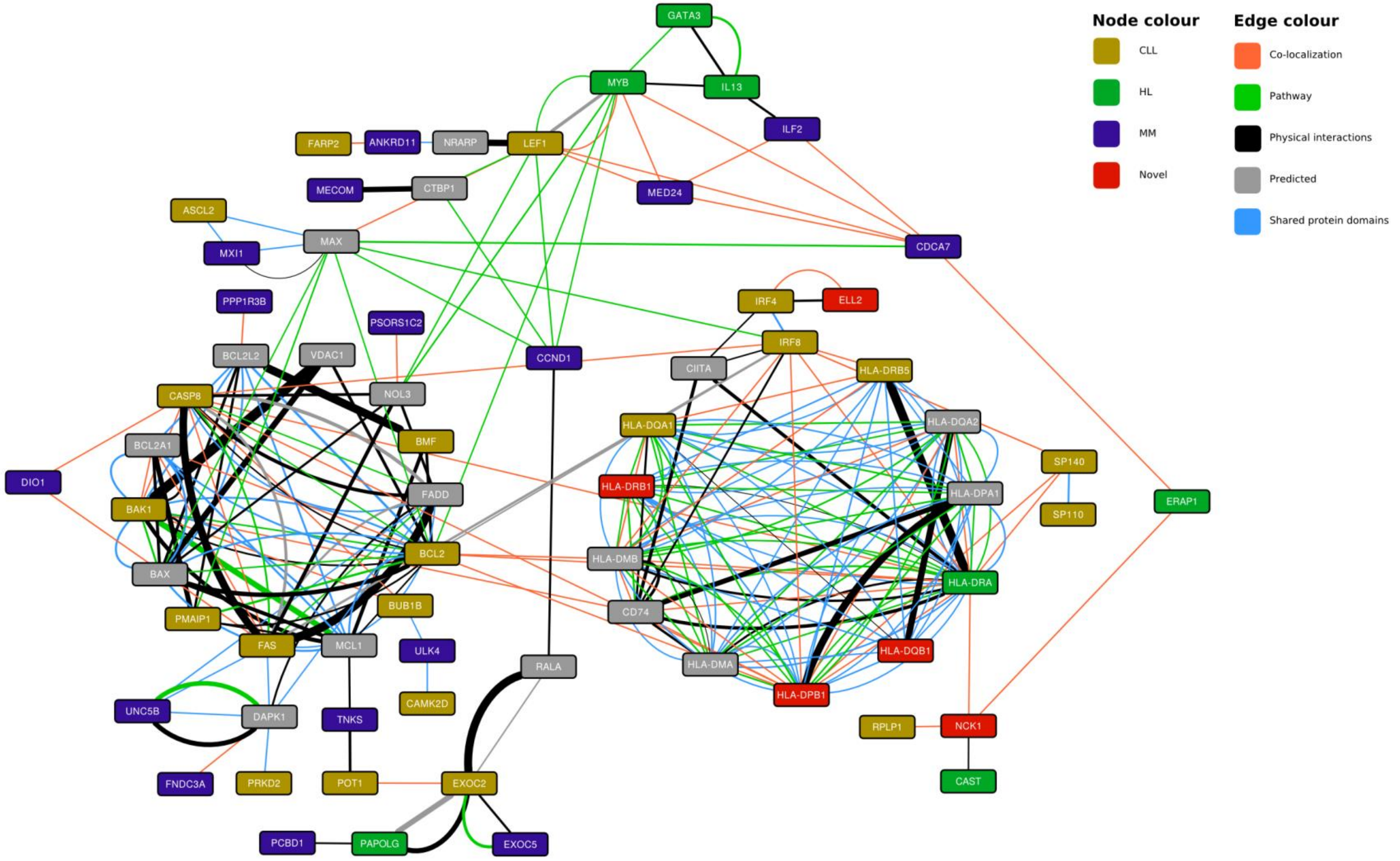

Supplement: Supplementary Data [file srep41071-s1.pdf]
